# Steatosis of indeterminate cause in a pediatric group: is it a primary mitochondrial hepatopathy?

**DOI:** 10.1590/S1516-31802011000400004

**Published:** 2011-05-05

**Authors:** Gustavo Henrique Silva, Gabriel Hessel, Kunie Iabiku Rabello Coelho, Cecília Amélia Fazzio Escanhoela

**Affiliations:** I PhD. Postgraduate student, Department of Pathology, Faculdade de Ciências Médicas, Universidade Estadual de Campinas (FCM-Unicamp), Campinas, São Paulo, Brazil.; II MD, PhD. Assistant professor, Department of Pediatrics, Faculdade de Ciências Médicas, Universidade Estadual de Campinas (FCM-Unicamp), Campinas, São Paulo, Brazil.; III MD, PhD. Assistant professor, Department of Pathology, Faculdade de Medicina de Botucatu, Universidade Estadual Paulista (FMB-Unesp), Botucatu, São Paulo, Brazil.; IV MD, PhD. Assistant professor, Department of Pathology, Faculdade de Ciências Médicas, Universidade Estadual de Campinas (FCM-Unicamp), Campinas, São Paulo, Brazil.

**Keywords:** Fatty liver, Mitochondria, liver, Anatomy, Infant, newborn, Metabolism, inborn errors, Fígado gorduroso, Mitocôndrias hepáticas, Anatomia, Recém-nascido, Erros inatos do metabolismo

## Abstract

**CONTEXT AND OBJECTIVE:**

In children, hepatic steatosis may be related to inborn errors of metabolism (IEMs) or to non-alcoholic fatty liver disease (NAFLD). The aim of this study was to assess and characterize steatosis of indeterminate cause through morphological and morphometric analysis of liver tissue.

**DESIGN AND SETTING:**

Cross-sectional study at the Departments of Pathology of Faculdade de Ciências Médicas, Universidade Estadual de Campinas (FCM-Unicamp) and Faculdade de Medicina de Botucatu, Universidade Estadual Paulista (FMB-Unesp).

**METHODS:**

Eighteen consecutive liver biopsies obtained from 16 patients of ages ranging from 3 months to 12 years and nine months that were inserted in a database in the study period were analyzed using optical microscopy and transmission electron microscopy. Through electron microscopy, the mitochondrial density and mean mitochondrial surface area were determined in hepatocytes. Ten patients ranging in age from 1 to 14 years were used as a control group.

**RESULTS:**

"Pure" steatosis was detected, unaccompanied by fibrosis or any other histological alteration. Microvesicular steatosis predominated, with a significant increase in mean mitochondrial surface area.

**CONCLUSION:**

Microvesicular steatosis may be related to primary mitochondrial hepatopathy, especially due to reduction of b-oxidation or partial stagnation of oxidative phosphorylation. For these reasons, this form of steatosis (which should not be called "pure") is likely to represent an initial stage in the broad spectrum of NAFLD. We have drawn attention to cases of steatosis in the pediatric group, in which the microvesicular form predominates, since this may be associated with mitochondrial disorders.

## RESUMO

**CONTEXTO E OBJETIVO:**

Em crianças, a esteatose hepática pode se relacionar a erros inatos do metabolismo (EIMs) ou à doença hepática gordurosa não-alcoólica (DHGNA). O objetivo deste estudo foi avaliar e caracterizar esteatose de causa indeterminada por meio de análises morfológica e morfométrica em tecido hepático.

**TIPO DE ESTUDO E LOCAL:**

Estudo transversal nos Departamentos de Patologia da Faculdade de Ciências Médicas da Universidade Estadual de Campinas (FCM-Unicamp) e Faculdade de Medicina de Botucatu da Universidade Estadual Paulista (FMB-Unesp).

**MÉTODOS:**

Foram utilizadas 18 biópsias hepáticas consecutivas obtidas de 16 pacientes com idade variando de 3 meses a 12 anos e 9 meses, inseridas num banco de dados no período do estudo, que foram analisadas por microscopia óptica e eletrônica. Na microscopia eletrônica, foi realizada determinação da densidade mitocondrial e da área superficial média das mitocôndrias nos hepatócitos. Dez pacientes com idade variando de 1 a 14 anos foram usados como grupo controle.

**RESULTADOS:**

Foi detectada esteatose "pura", não acompanhada por fibrose ou outra alteração histológica. Foi verificado que, na predominância de esteatose microvesicular, houve aumento significativo da área mitocondrial média.

**CONCLUSÃO:**

A esteatose microvesicular pode estar relacionada à hepatopatia mitocondrial primária, principalmente devido à redução na b-oxidação ou parcial estagnação da fosforilação oxidativa. Por essas razões, esta forma de esteatose (que não pode ser chamada de "pura") possivelmente represente uma fase inicial no amplo espectro da DHGNA. Chamamos a atenção para casos de esteatose no grupo pediátrico com predomínio da forma microvesicular, uma vez que pode haver associação com desordens mitocondriais.

## INTRODUCTION

In children, the presence of steatosis is generally related to inborn errors of metabolism (IEMs). The clinical presentation varies and includes changes to general health condition; neurological, digestive and respiratory disorders; rapid and progressive neurological deterioration; myopathy; cardiomyopathy; craniofacial dysmorphism etc. Some forms of the disease present essentially as liver disease with hepatomegaly, jaundice, vomiting, lethargy and abnormalities of liver function.

When these diseases are ruled out after clinical and laboratory investigation, non-alcoholic fatty liver disease (NAFLD) should be considered. This term, which was initially used for adults, has become more inclusive.^1^ It is currently used to describe all age groups, even children.^2,3^ In more recent years, several studies on childhood^4^ and adolescent hepatic steatosis in cases of NAFLD have been conducted, especially in relation to obese children,^5-13^ since the prevalence of NAFLD is higher in these types of children. In a pediatric group, the risk factors for NAFLD include obesity, insulin resistance and hypertriglyceridemia.^14^ The major features are a higher rate in male children, with serum alanine aminotransferase (ALT) levels that are usually higher than serum aspartate aminotransferase (AST) levels, and with hypertriglyceridemia and vague abdominal pain (which is usually the main reason for seeking clinical evaluation).^14-16^

According to Mandel et al.^17^ and Bioulac-Sage et al.,^18^ childhood microvesicular steatosis may be associated with mitochondrial abnormalities relating to increased numbers of organelles.^18,19^ There is also a correlation with respiratory chain defects and consequently impaired oxidative phosphorylation.^20,21^ When this type of abnormality occurs, transfer of electrons to oxygen molecules generates reactive oxygen species, thus producing superoxide anions and hydrogen peroxide.^22^ Furthermore, with stagnation of mitochondrial oxidative capacity, fatty acids accumulate in the cytosol, thus reaffirming the close relationship between steatosis and mitochondriopathy.

The microvesicular form alone may also be associated with some clinical conditions, such as Reye's syndrome, Jamaican vomiting sickness and congenital deficiency of b-oxidation.^23^ In an experimental study on pigs, it was observed that the regenerative capacity of the hepatocytes in macrovesicular steatosis was more effective than in microvesicular steatosis, thereby implying that the latter has a less favorable prognosis.^24^

The cases that were studied did not have any clinical association with any previously cited factor and the findings were correlated with clinical and laboratory data. There is a continuing debate on the advisability of performing liver biopsy, because of the risk of complications, high costs and lack of effective therapy. However, liver biopsy is the only precise diagnostic method, and it is essential for grading and staging the disease. Furthermore, it enables patient inclusion in clinical screening programs.^25,26^

## OBJECTIVE

The aim of our study was to evaluate and characterize "pure" steatosis in a pediatric group by means of morphological (optical and electron microscopy) and morphometric analysis.

## MATERIAL AND METHODS

All pediatric patients who were diagnosed as presenting morphological steatosis by means of optical microscopy and examination of liver fragments processed for transmission electron microscopy in the Department of Pathology of the School of Medical Sciences of Universidade Estadual de Campinas (FCM-Unicamp) and the Department of Pathology of Faculdade de Medicina de Botucatu (FMB) between 1993 and 2007 were retrospectively selected by means of a database search (specific records from liver biopsies). Cases in which the cause of steatosis was established (IEM, obesity, diabetes, inherited metabolic disorders, malnutrition, viral hepatitis, Wilson's disease, drug use and/or total parenteral nutrition) were subsequently excluded. Sixteen patients whose cause of steatosis could not be identified were selected. These patients’ ages ranged from three months to 12 years and nine months. Their heights ranged from 47 to 154.5 cm and their weights ranged from 3.15 to 51.50 kg. A review of all previously selected liver biopsies was undertaken: 18 samples were analyzed, because one patient underwent three biopsies (samples 12, 13 and 14).

The control group was composed of liver biopsies obtained from ten patients aged from one to 14 years who presented normal physical development. In this group, the indications for liver biopsy were similar to those in the study group, i.e. vague abdominal pain and/or minimal hepatomegaly associated with slightly and persistently elevated hepatic enzyme levels. The diagnoses for the control individuals were normal, confirmed either from optical or from electron microscopy findings. In all of these patients, the blood glucose levels were always normal. All the clinical data were obtained by consulting patient medical records. The present study was approved by the Research Ethics Committee of FCM-Unicamp (process no. 523/2004).

The liver specimens were obtained by means of percutaneous biopsy and were processed using optical microscopy and transmission electron microscopy (TEM). For optical microscopy, the specimens were fixed in 10% formalin and processed for embedding in Paraplast Plus. The 5 mm sections were stained with hematoxylin-eosin to semi-quantitatively analyze two parameters: a) intensity of total steatosis (macro and microvesicular); and b) estimated percentage of hepatocytes affected by microvesicular steatosis, compared with hepatocytes affected by macrovesicular steatosis. The intensity of total steatosis was classified from (1) to (4), as follows: (1) 5% to 25% of tissue affected by steatosis; (2) 25 to 50% of tissue affected; (3) 50 to 75% of tissue affected; and (4) more than 75% of tissue affected. To determine the percentage of hepatocytes affected by microvesicular steatosis, all cells present in the fragments analyzed were counted (cells with small-droplet steatosis and those with large-droplet steatosis) and then the percentages of each cell type were calculated. Specimens processed for TEM were fixed in a 2.5% glutaraldehyde solution, postfixed in a 1% osmium tetroxide solution and embedded in araldite resin. Ultrathin sections (70-80 nm) were observed under a LEO 906 (Zeiss) electron microscope. For each patient, a liver tissue block was analyzed to determine the mitochondrial surface area in 10 microscope fields at a magnification of 6,000 x and the mean mitochondrial density, by counting the number of mitochondria present in 10 hepatocytes. Since this was a retrospective study, identification of the liver acinus zone at the time of inclusion was not a cause for concern. The acinus zone could not be characterized in this study because of the small dimensions of the TEM blocks. However, hepatocytes were randomly selected in all cases, thereby minimizing the error relating to sampling.

The results obtained were compared with the mean values found in the control group of patients. The inclusion criteria for the control group were that these individuals should be under 14 years of age and present a liver biopsy that was diagnosed as normal (n = 10). In both analyses, image capture was analyzed with the aid of the TPS Dig 1.30 software. The difference in steatosis between the patients and controls was evaluated using analysis of variance (ANOVA) with log transformation. P values < 0.05 were arbitrarily taken to be significant ("acceptable error limit").

## RESULTS

All the samples were described as having fatty liver, without fibrosis and/or deposition in hepatocytes or Kupffer cells. For each sample evaluated, Table 1 shows the total steatosis values obtained by means of semiquantitative analysis and the microvesicular steatosis values obtained by means of estimated analysis. The total steatosis ranged from one to four and the microvesicular steatosis ranged from five to 95%. Considerable variation was found in the degree of steatosis, as well as in the percentage of microvesicular steatosis compared with macrovesicular steatosis. Because of this variation, the patients were allocated into two groups: the first group was composed of patients with predominantly (> 50%) microvesicular steatosis (n = 11); and the second group was composed of patients with predominantly (≤ 50%) macrovesicular steatosis (n = 7).

**Table 1.** Description of some parameters evaluated from the 16 patients studied (18 samples)

Group	Sample	Age	Sex	BMI (kg/m^2^)	AST (U/I)	ALT (U/I)	GGT (U/I)	Chol (mg/dl)	Trig (mg/dl)	Total steatosis	Microvesicular steatosis
Reference values					AST < 37 U/I	ALT < 40 U/I	GGT < 40 U/I	total cholesterol < 200 mg/dl	triglycerides < 120 mg/dl		
Macrovesicular	01	1y 11m	F	18.7	20	72	ni	134	46	4	40%
	03	11y	M	21.6	68	99	28	219	109	3	45%
	08	1y 2m	M	15.7	73	48	39	216	172	4	20%
	11	5m	M	14.6	87	386	554	131	347	4	25%
	12	4y 6m	M	17.5	47	48	20	ni	34	2	40%
	14	1y 1m	M	15.9	25	25	37	146	51	4	50%
	18	6m	F	13.5	119	204	585	133	476	4	5%
Microvesicular	02	10m	F	15.7	75	71	ni	216	1330	4	55%
	04	6y	F	14.3	304	104	340	103	516	3	70%
	05	3y 8m	F	14.9	24	17	15	172	54	1	70%
	06	1y 1m	M	17.0	75	50	384	144	152	3	70%
	07	5m	F	12.8	242	151	ni	ni	ni	3	75%
	09	3m	M	ni	14	19	212	182	421	3	70%
	10	4m	F	13.7	37	63	197	172	455	3	75%
	13	2y	M	16.5	26	22	50	156	54	4	60%
	15	10 m	M	15.8	47	53	31	105	87	4	80%
	16	11y 1m	M	14.7	20	18	ni	166	75	2	95%
	17	12y 9m	M	ni	39	63	257	281	286	3	75%

BMI = body mass index; ni = nothing included; y = years; m = months; F = female; M = male; AST = aspartate aminotransferase; ALT = alanine aminotransferase; GGT = gamma-glutamyl transpeptidase; Chol = total cholesterol; Trig = triglycerides.

Marked variation in hepatic enzyme values was observed. The AST levels ranged from 20 to 304 U/I; ALT from 17 to 386 U/I; and gamma-glutamyl transpeptidase (GGT) from 15 to 585 U/I. In Table 1, the biochemical parameters, weight and height of each patient are shown.

Analysis on the mitochondrial ultrastructure showed that the most frequent morphological abnormalities were megamitochondria, with or without crystalline inclusions, and a highly irregular mitochondrial shape, with degenerative changes (decreased electron density and loss of cristae).

**Figure 1.**
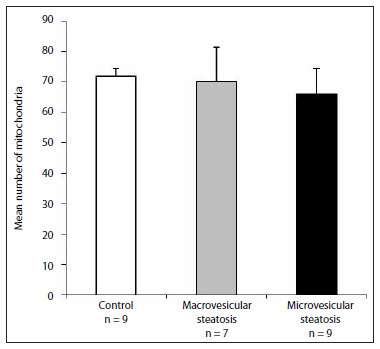
Mean mitochondrial density in the hepatocytes from patients with steatosis, compared with patients in the control group. Analysis of variance (ANOVA) with log transformation.

**Figure 2.**
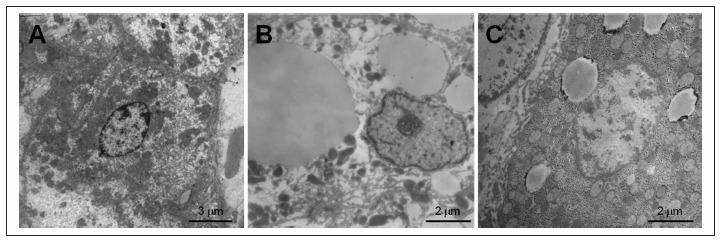
Electron micrographs showing differences in relation to mitochondrial density. A: Hepatocyte from a subject in the control group; B: Hepatocyte from a patient with macrovesicular steatosis; C: Hepatocyte from a patient with microvesicular steatosis.

Morphometric analysis on mitochondrial density showed that there were no significant differences between the control group, microvesicular steatosis group and macrovesicular steatosis group, as demonstrated in Figure 1. Images showing the differences in the quantities of mitochondria in the hepatocytes are presented in Figure 2.

**Figure 3.**
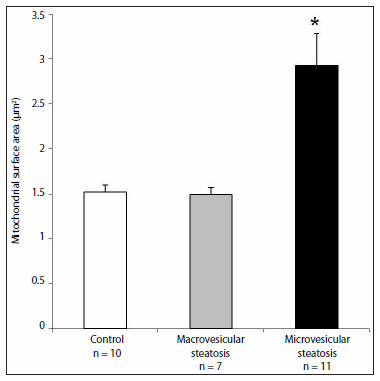
Mean mitochondrial surface area in the hepatocytes from patients with steatosis compared with patients in the control group. (*) represents a significant difference between the group with microvesicular steatosis, the control group and the group with macrovesicular steatosis. Analysis of variance (ANOVA) with log transformation (P < 0.05).

**Figure 4.**
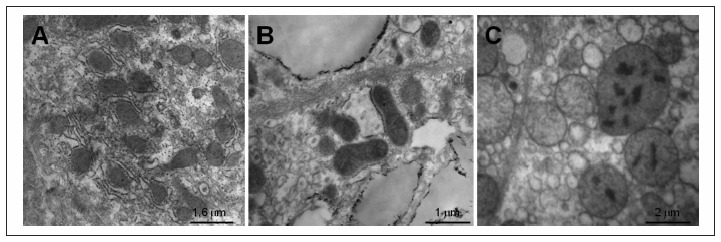
Electron micrographs showing differences in relation to mean mitochondrial surface area in the hepatocytes. A: Mitochondria in the hepatocytes from a subject in the control group; B: Mitochondria in the hepatocytes from a patient with macrovesicular steatosis; C: Mitochondria in the hepatocytes from a patient with microvesicular steatosis showing considerable dimensions (megamitochondria) and crystalline inclusions.

Analysis on the mean mitochondrial surface area showed that there was a significant difference between the microvesicular steatosis group and the control and macrovesicular steatosis groups. These results are presented in Figure 3 and images are shown in Figure 4.

## DISCUSSION

Since mitochondria are the sites for many metabolic processes (such as tricarboxylic acid production, fatty acid β-oxidation, synthesis of urea and other substances, and use of glucose and fatty acids as fuels for ATP synthesis), a disorder in any of these mechanisms may cause severe damage to the cell and thus to the tissue. Respiratory chain defects are not rare and are known to cause early liver failure in infancy,^21^ thereby leading to a change in fatty acid oxidation.^27^ Changes to mitochondrial β-oxidation may result in accumulation of fatty acids in the cytosol, and this is typically represented by microvesicular steatosis.^28,29^

In our study, a significant elevation in mean mitochondrial surface area was observed, and this proved to be substantial in the patients with predominantly microvesicular steatosis. In contrast, the mitochondria in the patients with predominantly macrovesicular steatosis were considered to be normal in size. These results corroborate the reports by Sherlock and Dooley^30^ indicating that microvesicular steatosis is of greater clinical importance and may progress in a more troubling manner.

Many other reports on the relationship between steatosis and mitochondrial alterations have been published in the literature. Studies by Sokol and Treem^27^ have reported that steatosis in neonatal liver failure is related to increased mitochondrial density, with an occasional increase in mitochondrial size. Mandel et al.^17^ observed mitochondrial abnormalities in the matrix and cristae, and also an increase in the total number of mitochondria, in cases of microvesicular steatosis. Bioulac-Sage et al.^18^ reported mitochondrial proliferation ("oncocytic" changes) in patients with microvesicular steatosis.

Despite an evident increase in mitochondrial size in patients with microvesicular steatosis, changes to mitochondrial density were not clearly expressed in this morphometric model. To explain this result, our suggestion is that the normal distribution of mitochondria in the cytosol becomes altered because of the existence of lipid vacuoles in hepatocytes. Changes to mitochondrial distribution then disrupt the regularity of the outcome, especially when comparing the results from patients with steatosis to those from the control group (in which there are no lipid vacuoles in the cell).

In our case study, 64% of the patients with microvesicular steatosis and 57% of the patients with macrovesicular steatosis showed an elevation in at least one hepatic serum aminotransferase. Some authors such as Rashid and Roberts^15^ and Molleston et al.^16^ have reported higher rises in AST than in ALT. However, this relationship is quite typical of alcoholic hepatitis, in which the AST/ALT ratio is greater than one.^2^ In our case study, only 39% of the patients with steatosis (of both types) presented elevated serum transaminase levels with an AST/ALT ratio greater than one. Elevated serum aminotransferase levels seem to show no direct relationship with either type of steatosis. Thus, no relationship with increased mitochondrial volume is found, since the enzyme levels vary widely, both in patients with predominantly microvesicular steatosis and in those with predominantly macrovesicular steatosis.

Another indication of compromised mitochondria is the frequent observation of megamitochondria, often showing crystalline inclusions. Such findings have been correlated with Wilson's disease^31^ and nonalcoholic steatohepatitis (NASH).^32^ More recently, it has been indirectly correlated with oxidative stress in NAFLD cases.^33,34^

The mechanisms that induce mitochondrial "hypertrophy" remain obscure. However, changes to the respiratory chain or to other mitochondrial metabolic enzymes, whether inborn or acquired, may lead to an increase in b-oxidation and partial stagnation of oxidative phosphorylation (OxP). We hypothesize that an increase in mitochondrial volume occurs as a consequence. As a result, the volumes of the inner membrane (and cristae) and inner membrane space (sites relating to b-oxidation and OxP) increase in an attempt to compensate for metabolic processes that were partially or totally interrupted. Taking into account the "two-hit" hypothesis proposed by Day and James^35^ to explain the pathogenesis of NAFLD, it is important to note that steatosis by itself does not cause the development of liver disease. However, it is a factor that can sensitize the liver to the damaging effects of a "second hit." Therefore, factors causing innocuous stress in healthy livers may lead to the development of NASH in steatotic livers.^36^

The results from this study have contributed substantially towards evaluations on childhood steatosis of indeterminate cause, drawing close attention to cases with predominantly microvesicular steatosis. No similar studies on pediatric groups have been published in the literature, and we would particularly like to highlight that the data on the control group in this study, which has also never previously been published, may be useful as a reference for conducting other electron microscopy studies on pediatric livers.

## CONCLUSION

It may be concluded from our results that changes to serum transaminases and the presence of macrovesicular steatosis in this pediatric group did not correlate with changes to mitochondrial density or size. In addition, increased mitochondrial size was probably a morphological expression relating to deranged beta-oxidation and partial stagnation of oxidative phosphorylation. This therefore supports the hypothesis that there may be a mild form of primary mitochondrial hepatopathy, which may then progress in a more worrisome manner, especially as a second hit.
